# The metabolic score of insulin resistance is positively correlated with bone mineral density in postmenopausal patients with type 2 diabetes mellitus

**DOI:** 10.1038/s41598-023-32931-8

**Published:** 2023-05-31

**Authors:** Peng Gu, Bin Pu, Qiao Xin, Dan Yue, LieLiang Luo, JiaSheng Tao, HaiShan Li, Ming Chen, MingHua Hu, XiaoRong Hu, XiaoHui Zheng, ZhanPeng Zeng

**Affiliations:** 1 Guangzhou University of Chinese Medicine, Guangzhou, Guangdong China; 2 The First Affiliated Hospital, Guangzhou University of Chinese Medicine, Guangzhou, China; 3 Jiangxi University of Chinese Medicine, Nanchang, Jiangxi China; 4 Southwest Medical University, Luzhou, Sichuan China

**Keywords:** Biomarkers, Endocrinology, Medical research

## Abstract

The prevalence of type 2 diabetes mellitus (T2DM) complicated with osteoporosis (OP) is increasing yearly. Early prevention, detection and treatment of OP are important in postmenopausal patients with T2DM. This study aimed to explore the correlation between insulin resistance and bone mineral density (BMD), and OP in postmenopausal patients with T2DM. In this study, postmenopausal patients with T2DM who visited our hospital from January 2021 to March 2022 were divided into the OP group (n = 91) and non-OP group (n = 119) according to whether they were complicated with OP or not. The general data of patients, BMD, blood routine, glucose metabolism, lipid metabolism, liver and kidney function indexes were collected, and the homeostatic model assessment for IR (HOMA-IR), the triglyceride-glucose (TyG) index and the metabolic score for IR (METS-IR) were calculated. A weighted multivariate linear regression model assessed the correlation between insulin resistance (IR) related indexes and lumbar spine, femoral neck, and hip BMD. A weighted logistic regression model assessed the odds ratios (ORs) and 95% confidence intervals (95% CIs) for the association between the IR-related indexes and OP risk. The nonlinear relationship was also evaluated by smooth curve fitting (SCF) and a weighted generalized additive model (GAM). Moreover, the Receiver-operating characteristics (ROC) curve was used to analyze the predictive efficiency of METS-IR in postmenopausal patients with T2DM with OP. HOMA-IR, TyG, and METS-IR in the OP group were lower than those in the non-OP group (all P < 0.05). Weighted multiple linear regression after adjusting covariates showed that METS-IR was positively correlated with the lumbar spine, femoral neck, and hip BMD (β_METS-IR_ = 0.006,0.005,0.005, all P < 0.001). The results of weighted Logistic regression and GAM showed that when METS-IR < 44.5, each unit of increased METS-IR value was associated with a decreased OP risk of 12% (P = 0.002). When METS-IR ≥ 44.5, there was no significant correlation between METS-IR and the risk of OP (OR = 1.00, P = 0.934). Similar trends were not observed in HOMA-IR and TyG. The ROC suggested helpful discriminative power of the METS-IR index for T2DM. We confirmed that METS-IR, as a novel alternative marker of IR, had a positive association with BMD in postmenopausal patients with T2DM, and METS-IR was a protective factor for OP in a specific range.

## Introduction

Osteoporosis (OP) is a disease that occurs and develops with age. It is characterized by the progressive reduction of bone mass and destruction of bone microstructure and is prone to fragility fractures^1^. About 1.5 million cases of osteoporotic fractures are reported worldwide annually, most of which are postmenopausal women^2^. It has resulted in a vast social and economic burden and has become a significant public health problem worldwide^3^. Patients with type 2 diabetes mellitus (T2DM) have a higher risk of OP and fragility fractures. Some studies have put forward the concept of diabetic osteoporosis and considered OP a significant complication of diabetes in the skeletal system^4,5^.

Insufficiency of insulin secretion caused by islet β cell dysfunction and insulin resistance (IR) or relative decrease are the leading causes of T2DM. Insulin can directly affect the function of osteoblasts and osteoclasts through insulin receptors or indirectly affect osteocyte metabolism by regulating vitamin D and parathyroid hormone levels^6–8^. Previous studies have confirmed that diabetes-related indicators (fasting insulin, FINS, fasting plasma glucose, FPG, glycated hemoglobin, HbA1c, insulin resistance, IR) were related to the risk of bone mineral density (BMD) and OP^9–12^. Among them, we are very interested in the relationship between IR and bone metabolism.

IR is a pathophysiological marker of OP and many other metabolic diseases. The euglycemic–hyperinsulinemic clamp technique is the gold standard for evaluating IR in humans^13^. However, this tool is unsuitable for large-scale epidemiological studies because of its invasive, and complicated nature. Therefore, massive studies have developed non-invasive and easy-to-operate assessment indicators of IR, such as the homeostatic model assessment for IR (HOMA-IR), the triglyceride-glucose (TyG) index and the metabolic score for IR (METS-IR)^13–15^.

Based on these perspectives, we innovatively put forward that IR-related indexes (HOMA-IR, TyG, METS-IR) may be related to BMD. Therefore, this study collected the serological indexes of postmenopausal patients with T2DM, analyzed the correlation between IR markers and OP, and predicted the diagnostic efficacy, aiming to find a novel, safe and simple indicator for the prevention and diagnosis of OP in postmenopausal patients with T2DM.

## Methods

### Study design and population

This was a single-center retrospective study. Postmenopausal patients with T2DM who received treatment in the first affiliated Hospital of Guangzhou University of Traditional Chinese Medicine from January 2021 to March 2022 were selected. All participants received standardized medication treatment for T2DM during hospitalization. Inclusion criteria: (a) hospitalized patients who were diagnosed with T2DM and had natural menopause, (b) patients with complete BMD and serological data. We excluded: (a) patients who have received anti-OP or oral hormone therapy that may affect bone metabolism for a long time (> 6 months) (n = 16); (b) patients with any acute infection or diabetic crisis (n = 8); (c) patients with severe heart failure, lung disease and hepatorenal insufficiency (n = 23); (d) patients with abnormal thyroid function, malignant tumor and other diseases affecting BMD (n = 22). This study was approved by the Ethics Committee of the first affiliated Hospital of Guangzhou University of Traditional Chinese Medicine (batch number: NO.K [2020] 102). The collected data does not contain any private information identified as individuals. The patients who participated in the trial volunteered to participate, fully informed consent to the trial process, and signed the informed consent form to understand the treatment plan fully. The study was conducted in accordance with the Helsinki Declaration.

### Clinical data

General data were obtained from the medical records of inpatients, which were collected separately by three researchers through the JiaHe medical record system. The patients' demographic data (age, height, and weight) and clinical features (history of previous and medication) were obtained through face-to-face interviews between residents and patients. Body mass index (BMI) is calculated by dividing weight by the square of height (kg/m^2^). From the laboratory examination on the second day of admission, we extracted information on serum calcium (Ca), serum phosphorus (P), total cholesterol (TC), triglyceride (TG), high-density lipoprotein cholesterol (HDL-C), low-density lipoprotein cholesterol (LDL-C), uric acid (UA), HbA1c, FINS, FPG, fasting C-peptide (FCP), glomerular filtration rate (eGFR), serum creatinine (SCr), alanine aminotransferase (ALT) and aspartate aminotransferase (AST). Blood samples were collected after ≥ 8 h of fasting and analyzed using Roche Covas701, Covas702 biochemical analyzer, Mindray BC-6800_A automatic five-classification blood cell analyzer.

### Assessment of insulin resistance

HOMA-IR and TyG index, and METS-IR are calculated as follows^13,15,16^:

HOMA-IR = [FINS (µU/mL) × FPG (mg/dL)/405].

TyG index = ln [TG (mg/dL) × FPG (mg/dL)/2].

METS-IR = ln [2 × FPG (mg/dL) + TG (mg/dL)] × BMI (kg/m^2^)/ln [HDL-C (mg/dL)].

### Assessment of BMD

The BMD of lumbar vertebrae L1-4, left femoral neck, and left hip were measured by dual-energy X-ray absorptiometry (Lunar company, American model: DPX- L type), and T values were recorded. According to the diagnostic criteria of OP put forward by the World Health Organization^17^, this study defined T < − 2.5 as the OP group, osteopenia (− 2.5 ≤ T ≤ − 1.0), and normal bone mass (T ≥ − 1.0) as the non-OP group. The baseline characteristics of all subjects were described by mean ± standard deviation (continuous variable) or rate (classified variable). T-test or X^2^ test was used for comparison between the two groups.

### Statistical analysis

The linear relationship between clinical indexes and BMD of all participants was analyzed using single-factor linear regression, and the indexes with the linear relationship were taken as covariates. The regression coefficient β value, P value, and the corresponding 95% confidence interval (CI) between HOMA-IR, TyG, METS-IR, and BMD were determined by weighted multiple linear regression analysis. The association between HOMA-IR, TyG, METS-IR, and OP risk of all participants was assessed using the unadjusted and adjusted weighted Logistic regression. The adjusted variables were selected from the binary Logistic regression analysis between covariates and OP. We use a generalized additive model (GAM) and smooth curve fitting (SCF) to address nonlinearity. In addition, the two-piecewise binary logistic regression model was used to explain the nonlinearity further. The diagnostic efficacy of METS-IR in predicting the occurrence of OP in postmenopausal patients with T2DM was assessed using Receiver-operating characteristics (ROC). Sensitivity refers to the percentage of OP patients who are positive by METS-IR; specificity refers to the percentage of non-OP patients who are negative by METS-IR. The optimal critical value is calculated by using the Youden index (Youden index = sensitivity–specificity). The range is between 0 to 1, and the higher the index is, the higher the prediction efficiency is. When Youden’s index is maximum, the corresponding value is the best threshold.

All analyses were conducted using R (version 4.0.3) and EmpowerStats software. The figures were generated using GraphPad Prism 9.0.0 (121). A double-tailed P-value < 0.05 was considered statistically significant in all analyses.

### Ethics approval and informed consent statement

The study was approved by the Institutional Ethics Committee of The First Affiliated Hospital, Guangzhou University of Chinese Medicine for retrospective analysis (ethics number: NO.K [2020] 102).

## Results

### Participant selection and baseline characteristics

**Figure 1:**
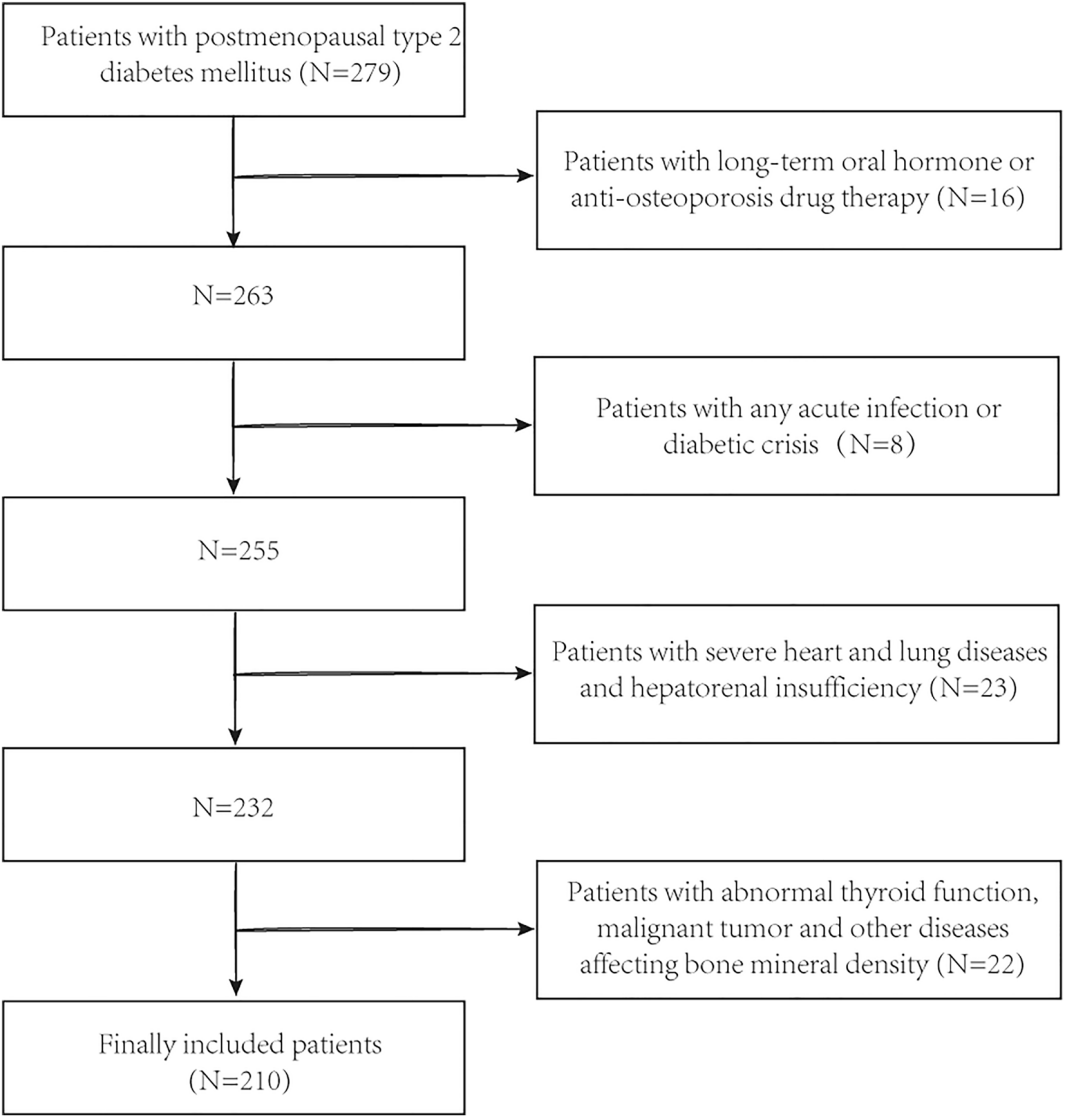
Flow chart of participants selection.

The study included 210 postmenopausal patients with T2DM treated in our center (Fig. 1), who were divided into the OP group (n = 91) and non-OP group (n = 119).

The baseline characteristics of selected participants were compared between the OP and non-OP groups (Table 1). The average age of patients in the OP group was higher than that in the non-OP group (68.74 years vs. 61.94 years, P < 0.001). Patients in the OP group were thinner (22.55 ± 3.22 kg/m^2^ vs. 24.16 ± 3.55 kg/m^2^, P = 0.001). The average BMD of lumbar vertebrae, femoral neck, and hip in the OP group were 0.82, 0.66, and 0.72 g/cm^3^, respectively, and those in the non-OP group was 1.09, 0.85, and 0.92 g/cm^3^, respectively. Serum P, FPG, FINS, FCP, TYG, HOMA-IR, and METS-IR in the OP group was significantly lower than in the non-OP group (all P < 0.05).

**Table 1:** Basic characteristics of the participants.

Characteristics	All participants (210)	OP (91)	Non-OP (119)	P
Age (years)	64.89 ± 8.28	68.74 ± 7.89	61.94 ± 7.33	< 0.001
BMI (kg/m^2^)	23.46 ± 3.50	22.55 ± 3.22	24.16 ± 3.55	0.001
Hypertension (%)				0.843
Yes	123 (58.57%)	54 (59.34%)	69 (57.98%)	
No	87 (41.43%)	37 (40.66%)	50 (42.02%)	
Serum calcium (mmol/L)	2.29 ± 0.11	2.28 ± 0.12	2.29 ± 0.10	0.571
Serum phosphorus (mmol/L)	1.23 ± 0.18	1.20 ± 0.17	1.25 ± 0.18	0.011
FPG (ug/dL)	133.73 ± 56.24	125.15 ± 51.64	140.30 ± 58.89	0.040
TC (ug/dL)	87.22 ± 21.05	85.82 ± 20.65	88.29 ± 21.37	0.303
TG (ug/dL)	30.16 ± 25.31	28.28 ± 21.41	31.60 ± 27.93	0.113
HDL-C (ug/dL)	22.23 ± 5.73	22.94 ± 6.42	21.69 ± 5.11	0.280
LDL-C (ug/dL)	56.15 ± 22.04	55.81 ± 25.00	56.41 ± 19.58	0.319
HbA1c (%)	8.48 ± 2.12	8.50 ± 2.25	8.46 ± 2.02	0.784
FINS (uiu/ml)	10.72 ± 11.79	8.10 ± 7.49	12.72 ± 13.93	0.004
FCP (ng/ml)	2.48 ± 3.23	2.03 ± 1.37	2.82 ± 4.09	0.023
SUA (umol/L)	325.41 ± 107.46	314.41 ± 105.83	333.82 ± 108.37	0.223
eGFR (ml/min)	77.64 ± 22.49	74.38 ± 23.58	80.14 ± 21.38	0.061
SCR (mg/dL)	67.20 ± 27.53	69.92 ± 31.07	65.13 ± 24.40	0.454
AST(u/L)	18.96 ± 13.74	17.96 ± 7.48	19.72 ± 17.04	0.706
ALT(u/L)	19.05 ± 14.93	18.42 ± 14.94	19.54 ± 14.97	0.237
TYG	7.36 ± 0.74	7.23 ± 0.77	7.46 ± 0.70	0.034
HOMA-IR	3.62 ± 4.40	2.77 ± 3.45	4.27 ± 4.92	0.001
METS-IR	43.40 ± 8.50	41.02 ± 8.54	45.23 ± 8.03	< 0.001
Lumbar spine BMD(g/cm^3^)	0.97 ± 0.19	0.82 ± 0.10	1.09 ± 0.15	< 0.001
Femoral neck BMD(g/cm^3^)	0.77 ± 0.15	0.66 ± 0.08	0.85 ± 0.13	< 0.001
Hip BMD(g/cm^3^)	0.84 ± 0.16	0.72 ± 0.10	0.92 ± 0.13	< 0.001

*OP* Osteoporosis, *BMI* body mass index, *FPG* fasting plasma glucose, *TC* total cholesterol, *TG* triglyceride, *HDL-C* high-density lipoprotein cholesterol, *LDL-C* low-density lipoprotein cholesterol, *HbA1c* glycated hemoglobin, *FINS* fasting insulin, *FCP* fasting C-peptide, *SUA* serum uric acid, *eGFR* glomerular filtration rate, *SCr* serum creatinine, *ALT* alanine aminotransferase, *AST* aspartate aminotransferase, *HOMA-IR* homeostatic model assessment for IR, *TyG* triglyceride-glucose, *METS-IR* metabolic score for IR, *BMD* bone mineral density.

### Associations of HOMA-IR, TyG, METS-IR with BMD

Univariate correlation analysis showed that age, HDL-C, and BMD were negatively correlated (all P < 0.05); BMI, serum Ca, serum P, FPG, FINS, SUA, eGFR, TYG, HOMA-IR, METS-IR were positively correlated with BMD (all P < 0.05) (Table 2).

**Table 2:** Correlation analysis between each index and BMD.

Index	Lumbar Spine BMD		Femoral neck BMD		Hip BMD	
	β	P-value	β	P-value	β	P-value
Age (years)	− 0.008	< 0.001	− 0.009	< 0.001	− 0.008	< 0.001
BMI (kg/m^2^)	0.016	< 0.001	0.008	0.004	0.012	< 0.001
Hypertension (%)	0.009	0.730	− 0.03	0.119	− 0.016	0.463
Serum calcium (mmol/L)	0.146	0.231	0.080	0.416	0.218	0.032
Serum phosphorus (mmol/L)	0.194	0.007	0.096	0.098	0.152	0.012
FPG (ug/dL)	0.000	0.105	0.000	0.023	0.000	0.011
TC (ug/dL)	0.000	0.761	0.000	0.700	0.000	0.513
TG (ug/dL)	0.001	0.110	0.000	0.276	0.001	0.102
HDL-C (ug/dL)	− 0.005	0.022	− 0.004	0.035	− 0.005	0.010
LDL-C (ug/dL)	− 0.000	0.418	− 0.000	0.723	− 0.000	0.748
HbA1c (%)	− 0.000	0.954	− 0.003	0.569	− 0.000	0.935
FINS (uiu/ml)	0.003	0.010	0.002	0.036	0.003	0.003
FCP (ng/ml)	0.007	0.083	0.004	0.270	0.006	0.078
SUA (umol/L)	0.000	0.007	0.000	0.231	0.000	0.025
eGFR (ml/min)	− 0.000	0.990	0.001	0.007	0.001	0.112
SCR (mg/dL)	0.000	0.601	− 0.001	0.172	− 0.001	0.770
AST (u/L)	0.001	0.321	0.001	0.230	0.001	0.281
ALT (u/L)	0.001	0.538	0.001	0.413	0.001	0.338
TYG	0.044	0.011	0.040	0.004	0.050	0.001
HOMA-IR	0.007	0.013	0.005	0.020	0.008	0.001
METS-IR	0.007	< 0.001	0.005	< 0.001	0.006	< 0.001

*BMD* bone mineral density, *BMI* body mass index, *FPG* fasting plasma glucose, *TC* total cholesterol, *TG* triglyceride, *HDL-C* high-density lipoprotein cholesterol, *LDL-C* low-density lipoprotein cholesterol, *HbA1c* glycated hemoglobin, *FINS* fasting insulin, *FCP* fasting C-peptide, *SUA* serum uric acid, *eGFR* glomerular filtration rate, *SCr* serum creatinine, *ALT* alanine aminotransferase, *AST* aspartate aminotransferase, *HOMA-IR* homeostatic model assessment for IR, *TyG* triglyceride-glucose, *METS-IR* metabolic score for IR.

Weighted multiple linear regression showed that METS-IR was still significantly positively correlated with lumbar vertebrae, femoral neck, and hip BMD after adjusting the covariates (β = 0.006, 0.005, 0.005 respectively, P < 0.001). However, no correlation was observed between HOMA-IR, TyG, and BMD (Table 3).

**Table 3:** Weighted multiple linear regression after adjusting covariates.

	Lumbar spine BMD		Femoral neck BMD		Hip BMD	
	β	P-value	β	P-value	β	P-value
TyG^a^	0.006	0.743	0.012	0.401	0.014	0.329
HOMA-IR^b^	0.003	0.237	0.003	0.166	0.004	0.050
METS-IR^c^	0.006	< 0.001	0.005	< 0.001	0.005	< 0.001

*BMD* bone mineral density, *HOMA-IR* homeostatic model assessment for IR, *TyG* triglyceride-glucose, *METS-IR* metabolic score for IR.

^a^adjusted for: age, body mass index, fasting insulin, serum calcium, serum phosphorus, high-density lipoprotein cholesterol and serum uric acid.

^b^adjusted for: age, body mass index, serum calcium, serum phosphorus, high-density lipoprotein cholesterol and serum uric acid.

^c^adjusted for: age, fasting insulin, serum calcium, serum phosphorus and serum uric acid.

### Associations of HOMA-IR, TyG, METS-IR with OP

Binary Logistic regression analysis showed that age was an independent risk factor for OP in postmenopausal patients with T2DM (OR = 1.125, P < 0.05). BMI, serum P, FINS, FCP, HOMA-IR, TyG, and METS-IR were independent protective factors (OR = 0.866, 0.187, 0.956, 0.818, 0.642, 0.906, and 0.938, respectively; P < 0.05) (Table 4).

**Table 4:** Correlation analysis between each index and OP.

Index	OP		Index	OP	
	OR	P-value		OR	P-value
Age (years)	1.125	< 0.001	ALT (u/L)	0.995	0.590
BMI (kg/m^2^)	0.866	0.001	SUA (umol/L)	0.998	0.196
Hypertension (%)	1.058	0.843	eGFR (ml/min)	0.989	0.067
Serum calcium (mmol/L)	0.477	0.579	SCR (mg/dL)	1.006	0.217
Serum phosphorus (mmol/L)	0.187	0.040	FINS (uiu/ml)	0.956	0.009
FPG (ug/dL)	0.995	0.056	FCP (ng/ml)	0.818	0.048
TC (ug/dL)	0.994	0.399	HbA1c (%)	1.009	0.887
TG (ug/dL)	0.994	0.360	TyG	0.642	0.026
HDL-C (ug/dL)	1.039	0.118	HOMA-IR	0.906	0.021
LDL-C (ug/dL)	0.999	0.845	METS-IR	0.938	< 0.001
AST (u/L)	0.989	0.374			

*OP* Osteoporosis, *BMI* body mass index, *FPG* fasting plasma glucose, *TC* total cholesterol, *TG* triglyceride, *HDL-C* high-density lipoprotein cholesterol, *LDL-C* low-density lipoprotein cholesterol, *HbA1c* glycated hemoglobin, *FINS* fasting insulin, *FCP* fasting C-peptide, *SUA* serum uric acid, *eGFR* glomerular filtration rate, *SCr* serum creatinine, *ALT* alanine aminotransferase, *AST* aspartate aminotransferase, *HOMA-IR* homeostatic model assessment for IR, *TyG* triglyceride-glucose, *METS-IR* metabolic score for IR.

**Figure 2:**
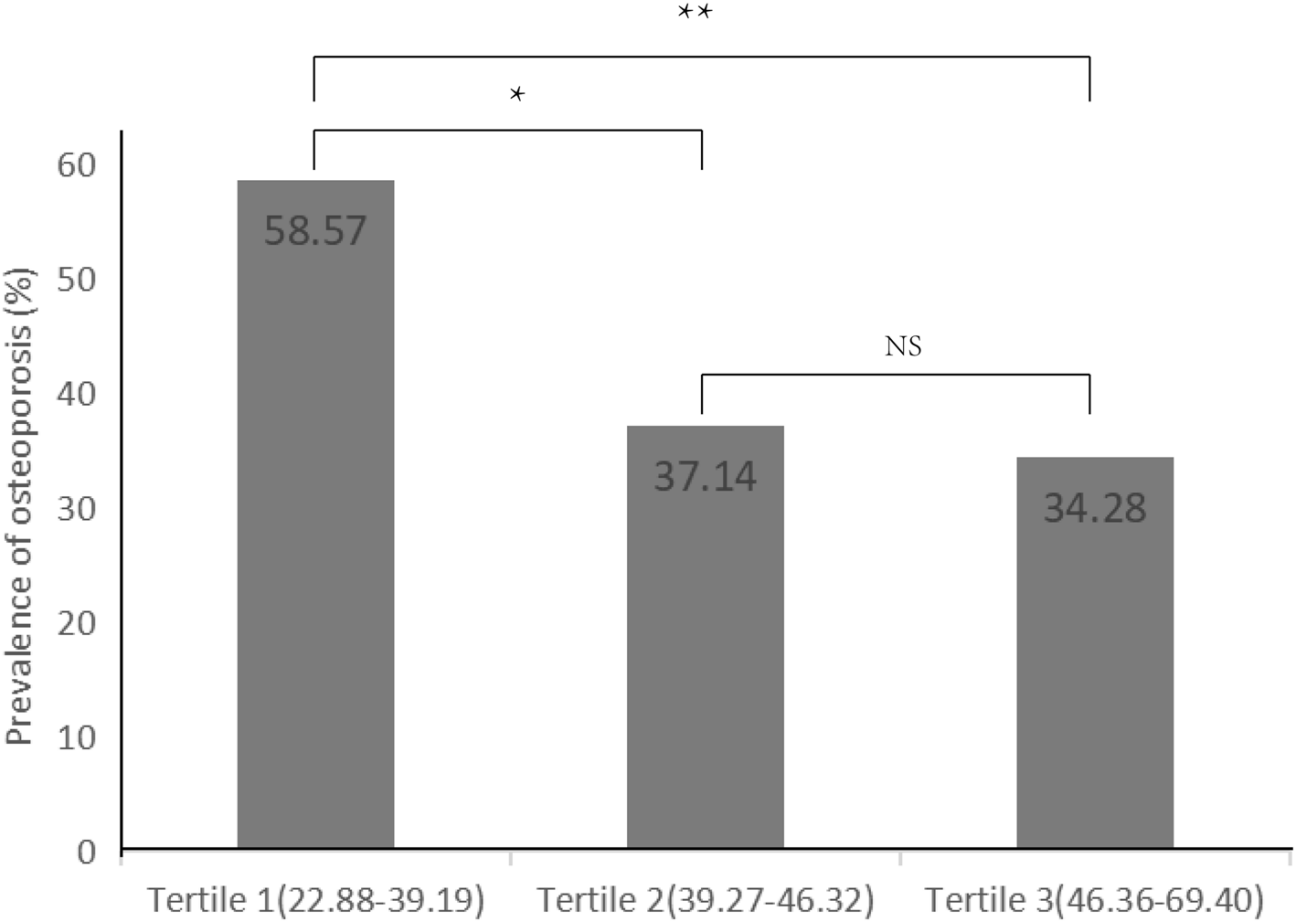
Prevalence of OP according to METS-IR index tertiles in postmenopausal patients with T2DM. The x-axis represents METS-IR values divided into trisections. The y-axis represents the prevalence of osteoporosis. Significant differences between groups are indicated by *(P = 0.011) and ** (P = 0.004), and NS indicates no significant difference (P > 0.05). *OP* Osteoporosis, *METS-IR* metabolic score for IR, *T2DM* type 2 diabetes mellitus.

Weighted Logistic regression analysis showed that METS-IR was still a protective factor for OP in postmenopausal patients with T2DM after adjusting covariates (OR = 0.940, P < 0.05), but no correlation was observed between HOMA-IR, TyG, and OP (Table 5). To further confirm our conclusion, we sorted the METS-IR values of the participants from small to large and then divided them into three parts according to the number of people. The prevalence rate of OP was 58.57%, 37.14%, and 34.29%, respectively, and there was a significant difference among the three groups (P = 0.007) (Fig. 2).

**Table 5:** Multivariate logistics regression after adjusting covariates.

Index	After adjustment	
	OR (95% CI)	P
TyG^**a**^	0.901 (0.571, 1.423)	0.655
HOMA-IR^**b**^	0.946 (0.855, 1.047)	0.285
METS-IR^**c**^	0.940 (0.900, 0.981)	0.004

*HOMA-IR* homeostatic model assessment for IR, *TyG* triglyceride-glucose, *METS-IR* metabolic score for IR.

^a^adjusted for: age, body mass index, fasting insulin, fasting C-peptide and serum phosphorus.

^b^adjusted for: age, body mass index, fasting C-peptide and serum phosphorus.

^c^adjusted for: age, fasting insulin, fasting C-peptide and serum phosphorus.

### Curve fitting and threshold effect analysis

**Figure 3:**
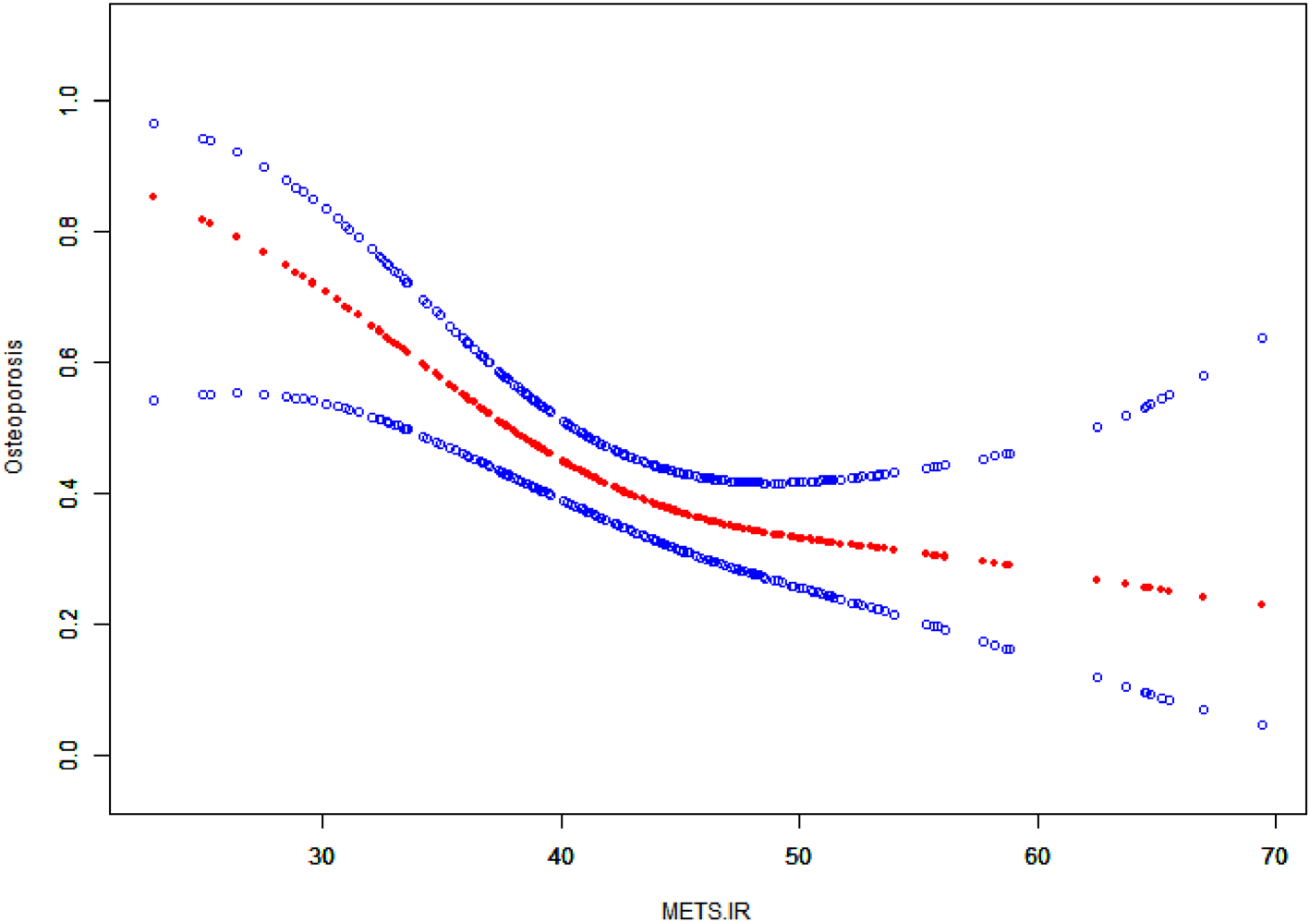
The non-linear relationship between METS-IR and incident of OP in postmenopausal patients with T2DM. Red line represents the smooth curve fit between variables. Blue lines represent the 95% CI of the fit. Adjust for: age, FINS, FCP and serum P. *METS-IR* metabolic score for IR, *OP* Osteoporosis, *T2DM* type 2 diabetes mellitus, *CI* confidence interval, *FINS* fasting insulin, *FCP* fasting C-peptide, *P* phosphorus.

After adjusting for age, FINS, FCP, and serum P, the results of GAM and SCF showed that the risk of osteoporosis changed with the increase of METS-IR value. It changed considerably initially, and after reaching a specific METS-IR value, the change of OP risk became smooth and showed a piecewise linear relationship (Fig. 3). By observing the fitting curve, we set the inflection point to 44.5 and used the two-piecewise logical regression model to evaluate the threshold effect of the fitting curve. The log-likelihood ratio test of METS-IR at inflection point 44.5 was statistically significant (P = 0.042), indicating that the two-piecewise regression model was suitable to describe the relationship between METS-IR and OP. When METS-IR < 44.5, each unit of increased METS-IR value was associated with a decreased OP risk of 12% (P = 0.002); When METS-IR ≥ 44.5, there was no significant correlation between METS-IR and the risk of OP (OR = 1.00, P = 0.934) (Table 6).

**Table 6:** Nonlinear Relationship Between METS-IR and OP.

	OR (95% CI)	P-value
Model I: univariate linear regression	0.94 (0.90, 0.98)	0.005
Model II: two-piecewise regression model		
Inflection point (K)	44.5	
< K point effect 1	0.88 (0.81, 0.95)	0.002
> K point effect 2	1.00 (0.93, 1.08)	0.934
Log-likelihood ratio test		0.042

Adjusted for: age, fasting insulin, fasting C-peptide and serum phosphorus.

*METS-IR* metabolic score for IR, *OP* Osteoporosis, *OR* odds ratio.

### Predictive efficacy of METS-IR on OP

**Figure 4:**
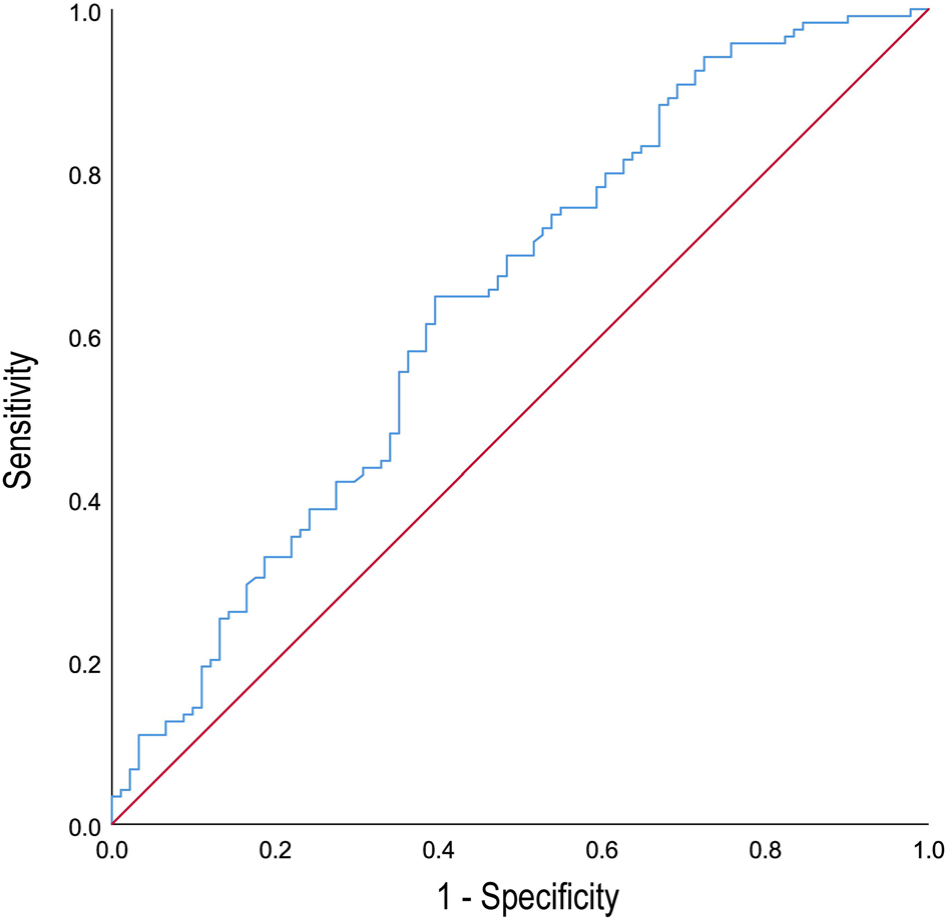
Receiver-operating characteristics (ROC) curves of the METS-IR index. Red line represents the ROC curve of invalid model. Blue line represents the ROC curve of METS-IR model.

The ROC curve showed that the area under the curve, sensitivity, and specificity of METS-IR in predicting the occurrence of OP in postmenopausal patients with T2DM were 0.639, 64.7%, and 60.4%, respectively, and the best cutoff value was 42.35 (Fig. 4).

## Discussion

This study provides new findings on the relationship between METS-IR, and BMD and OP in postmenopausal patients with T2DM. The METS-IR in the OP group was significantly lower than in the non-OP group. After adjusting the confounding factors, each unit of increased METS-IR value was associated with increased lumbar vertebrae, femoral neck, and hip BMD 0.006 g/cm^3^, 0.005 g/cm^3^, and 0.005 g/cm^3^, respectively (all P < 0.05). When METS-IR < 44.5, each unit of increased METS-IR value was associated with a decreased OP risk of 12%; When METS-IR ≥ 44.5, there was no significant correlation between METS-IR and the risk of OP. In addition, METS-IR has a certain predictive value for the risk of OP in postmenopausal patients with T2DM. In summary, it is helpful to measure and calculate METS-IR as an objective index to evaluate the risk of OP in the diagnosis and treatment of postmenopausal patients with T2DM.

Early diagnosis and risk assessment of OP in postmenopausal patients with T2DM is essential. In recent years, with the improvement of people's living standards and the change in living habits, the prevalence of OP in T2DM patients has increased yearly. Especially in postmenopausal women, due to the decrease of estrogen in the body, osteoclasts' inhibition, bone resorption enhanced, and massive bone loss led to the prevalence of OP significantly increasing^18^. At present, the clinical diagnosis of OP is mainly through dual-energy X-ray absorptiometry. The risk of OP can be evaluated by bone turnover markers, HDL-C, and BMI. Dual-energy X-ray is relatively expensive, has radiation and can only reflect the static, and local BMD of the patient^19^. Moreover, it is easy to underestimate the fracture risk in T2DM patients simply considering BMD alone^20^. Detecting bone turnover markers takes a long time, and many primary healthcare facilities lack relevant detection equipment. Using laboratory indexes such as HDL-C and BMI^21^ alone to predict the risk of OP has low sensitivity and specificity. Therefore, it is crucial to explore a more simple, economical, and accurate method to predict the risk of OP in postmenopausal T2DM.

IR is a state in which insulin is ineffective in peripheral tissues, leading to hyperinsulinemia and impaired lipid and glucose homeostasis. Among various methods for evaluating IR, the gold standard is the euglycemic–hyperinsulinemic clamp technique^22^, but this invasive method is unsuitable for the large-scale population. Some non-insulin indicators, such as TyG and METS-IR, combined with various serum biochemical indicators to evaluate IR, have attracted more and more attention. TyG is calculated from TG and FPG^15^, and METS-IR is calculated from HDL-C, TG, FPG, and BMI^13^. It is stated that these parameters are a non-insulin-based alternative to insulin-based methods to quantify peripheral insulin sensitivity. These indicators are easy to measure and calculate, so they are widely used in epidemiological studies and compared with traditional IR indicators. Cho et al.'s study^23^ of 1145 middle-aged people in Korea found that individuals with a high TyG correlation index are likelier to experience coronary artery calcification. Compared with HOMA-IR, TyG correlation index can better predict the progression of coronary artery calcification. In a study of 4,986 Korean adults, Lee et al.^24^ found that the TyG index has a better predictive power for NAFLD compared with HOMA-IR. A large cross-sectional study of 21,082 participants by Chen et al.^25^ found that the increase in METS-IR index was associated with a higher incidence of asthma and an earlier age of first asthma in American adults. Han et al.^26^ found a positive correlation between METS-IR and serum ferritin in a cross-sectional study of 4182 American women. This correlation was evident among participants ≥ 40 years old. Yoon et al.^27^ found that METS-IR was highly correlated with metabolic syndrome and cardiac metabolic risk, and METS-IR had better predictive value for ischemic heart disease than metabolic syndrome. Similarly, many studies have confirmed the correlation between IR and BMD, but the results are inconsistent, and we have not found any research on METS-IR and BMD. A cross-sectional study of postmenopausal women in Tunisia by Cherif et al.^28^ found that HOMA-IR was positively correlated with BMD of the left femur and total hip. Yoon et al.^29^ found that the TyG index negatively correlated with femoral neck BMD in non-diabetic men and postmenopausal women over 50 in a cohort study of 4810 non-diabetic Koreans. Zhou et al.^12^ found that the increase in HOMA-IR level was related to the increase of hip BMD in 7,170 American adults, but no causal relationship was found between IR and BMD in a Mendelian randomized study of European adults. In addition, numerous studies^30–33^ have proved that the indexes used to calculate METS-IR are significantly correlated with BMD. Therefore, this study collected serological indicators of postmenopausal patients with T2DM and evaluated the correlation between METS-IR and OP for the first time. The results showed that there was a significant positive correlation between METS-IR and BMD, and METS-IR was the protective factor of OP in postmenopausal patients with T2DM. However, we found that TyG and HOMA-IR had no significant correlation with BMD and OP.

These contradictory results may be due to different study populations or different assessment methods of IR. Based on the population of this study (postmenopausal patients with T2DM) and the IR assessment method (METS-IR), we believe that the possible mechanism of METS-IR affecting BMD and OP is as follows. Firstly, IR promotes insulin secretion, and hyperinsulinemia leads to an increase in BMD. Insulin can promote osteoblast proliferation, inhibit osteoclast activity, and act as an anabolic agent in bones^34^. In the state of IR, insulin secretion increases to compensate for the resistance of skeletal muscle, adipose tissue, and liver to insulin, which leads to hyperinsulinemia. Therefore, IR can promote insulin secretion and further increase bone mass. In addition, the synergistic effect of excessive insulin and other synthetic metabolic hormones (parathyroid hormone, insulin-like growth factor) can also lead to BMD increase^7,35^. Secondly, IR may further affect bone metabolism by affecting inflammatory response and estrogen levels. Wang et al.^36^ speculated that the relationship between IR and OP may not be linear and have a threshold effect. Our results confirm this view. In postmenopausal women with T2DM, when METS-IR < 44.5, the higher the IR, the lower the risk of OP. When METS-IR ≥ 44.5, the higher the IR, the greater the risk of OP. The reason may be that with the development of diabetes, the increase of pro-inflammatory cytokines and oxidative stress and the decrease of estrogen level has adverse effects on bone health, eliminating the protective effect of IR on the bone^37,38^. Finally, previous studies on IR and BMD mostly used TyG and HOMA-IR as evaluation indicators^12,23,24^. Our results showed that the BMD of lumbar vertebrae, femoral neck, and hip increased with the increasing TyG, HOMA-IR, and METS-IR in postmenopausal patients with T2DM. However, the association between TyG, HOMA-IR, and BMD lost significance after adjusting BMI. Napoli et al.^39^ found similar results in a prospective study of 2398 non-diabetic elderly. We think that compared with METS-IR, TyG, and HOMA-IR ignore the effects of BMI and other lipid types on bone metabolism. METS-IR is more comprehensive in evaluating metabolic status and is recognized as an effective index for IR estimation in the Chinese population^40–43^. Some chronic disease studies have also confirmed this view^13,27,44^.

The main advantage of this study is that METS-IR is used for the first time to evaluate the correlation of BMD and OP risk in postmenopausal patients with T2DM, which opens a new direction for the study of the correlation between IR and OP. To a certain extent, it can provide a breakthrough point for expanding OP-related predictive biological indicators and the screening, prevention, and treatment of OP in primary healthcare facilities. Despite the efforts made in this study, there are still some limitations. Firstly, this was a single-center cross-sectional study in which the sample size was small and the METS-IR was not repeatedly evaluated. The effectiveness of METS-IR changes over time in predicting OP risk was not obtained in postmenopausal patients with T2DM. Secondly, the population of this study is Chinese postmenopausal T2DM patients, and there are geographical and ethnic restrictions. The study results cannot be applied to healthy people or other races. Finally, the use of hypoglycemic drugs in inpatients was not collected in this study, so we cannot rule out the bias of hypoglycemic drugs on our results by affecting lipid metabolism. Therefore, large-scale, multicenter, high-level evidence-based research is still needed to confirm the relationship between METS-IR and OP in different populations.

## Conclusions

We confirmed that METS-IR, as a novel alternative marker of IR, had a positive association with BMD in postmenopausal patients with T2DM, and METS-IR was a protective factor for OP in a specific range. Therefore, we cautiously suggest that the risk of OP may need to be evaluated when the METS-IR decreases in postmenopausal patients with T2DM.

## Data Availability

All data generated or analyzed during this study are available from the corresponding author upon reasonable request.

## Abbreviations

T2DM: Type 2 diabetes mellitus
BMD: Bone mineral density
OP: Osteoporosis
IR: Insulin resistance
FINS: Fasting insulin
FPG: Fasting plasma glucose
HbA1c: Glycated hemoglobin
HOMA-IR: Homeostatic model assessment for IR
TyG: Triglyceride-glucose
METS-IR: Metabolic score for IR
BMI: Body mass index
Ca: Calcium
P: Phosphorus
TC: Total cholesterol
TG: Triglyceride
HDL-C: High-density lipoprotein cholesterol
LDL-C: Low-density lipoprotein cholesterol
SUA: Serum uric acid
FCP: Fasting C-peptide
eGFR: Glomerular filtration rate
SCr: Serum creatinine
ALT: Alanine aminotransferase
AST: Aspartate aminotransferase
ROC: Receiver-operating characteristics
GAM: Generalized additive model
SCF: Smooth curve fitting
OR: Odds ratio
CI: Confidence interval
